# Transmembrane potential, an indicator in situ reporting cellular senescence and stress response in plant tissues

**DOI:** 10.1186/s13007-023-01006-0

**Published:** 2023-03-21

**Authors:** Hai Liu, Yufei Li, Ting Peng, Shaowu Xue

**Affiliations:** 1 College of Life Science and Technology, Hubei Hongshan Laboratory, Huazhong Agricultural University, Wuhan, 430070 China; 2 College of Agriculture, Guizhou University, Guiyang, 550025 China

**Keywords:** Transmembrane potential, Senescence signal, Hyperpolarization, Depolarization, Abiotic stress, Organelles

## Abstract

**Background:**

Plant cells usually sustain a stable membrane potential due to influx and/or efflux of charged ions across plasma membrane. With the growth and development of plants, different tissues and cells undergo systemic or local programmed decline. Whether the membrane potential of plasma membrane could report senescence signal of plant tissues and cells is unclear.

**Results:**

We applied a maneuverable transmembrane potential (TMP) detection method with patch-clamp setup to examine the senescence signal of leaf tissue cells in situ over the whole life cycle in *Arabidopsis thaliana*. The data showed that the TMPs of plant tissues and cells were varied at different growth stages, and the change of TMP was higher at the vegetative growth stage than at the reproductive stage of plant growth. The distinct change of TMP was detectable between the normal and the senescent tissues and cells in several plant species. Moreover, diverse abiotic stimuli, such as heat stress, hyperpolarized the TMP in a short time, followed by depolarized membrane potential with the senescence occurring. We further examined the TMP of plant chloroplasts, which also indicates the senescence signal in organelles.

**Conclusions:**

This convenient TMP detection method can report the senescence signal of plant tissues and cells, and can also indicate the potential of plant tolerance to environmental stress.

**Supplementary Information:**

The online version contains supplementary material available at 10.1186/s13007-023-01006-0.

## Background

Senescence is a natural process that almost all living things will encounter. As the body's functions decline, individuals gradually tend to die [1, 2]. Additionally, a changeable environment and the interference of viruses influence the occurrence of this phenomenon [3–5]. In order to better maintain the healthy state of the body, it is particularly important to monitor the occurrence of senescence. There are many ways to monitor the fettle of tissues and cells in humans and animals [6–8], but monitoring senescence signals in plants appeared to be unfrequent. At present, the detectable indicators of plant senescence include decreased photosynthetic rate [9–11], abrupt changes in respiration rate [12], reduced synthesis and accelerated degradation of organics (sugar, protein and nucleic acid) [13–15], increased hydrolase activity [16], increased content of plant senescence-related hormones (abscisic acid and ethylene) [17–19] and redox imbalance causing reactive oxygen species to burst [20], etc. However, most of these detection methods are tedious to perform and cannot reflect the biological condition in vivo.

The plasma membranes of animals and plants maintain a certain transmembrane potential (TMP), usually positive on the outside and negative on the inside [21, 22]. The TMP is generated from the independent frequent exchange of charged ions across the cell membrane and the impermeability of macromolecular substances [22, 23]. The stable TMP can sustain the normal cellular metabolism, thereby maintaining the normal growth of individuals [24, 25]. Sudden changes in TMP can cause an anti-stress response in cells [26, 27]. Hyperpolarization occurs when cells are stimulated, *i.e.,* the TMP tends to be more negative; or depolarization occurs, *i.e.,* the TMP changes to be less negative [25, 28]. This provides a theoretical basis for directly monitoring the state of living cells.

Whether cellular TMP of plant tissues can be detected by simple means, so as to monitor the condition of plant tissues and cells, and to indicate the degree of senescence, is a practical problem that we hope to explore. In the present work, by using the patch-clamp device and referring to the microelectrode [29–32], we tried a more convenient method for detecting tissue TMP, and applied it to detect the activity of plant tissues and cells. We were surprised to find the TMP of plants in different growth stages showing regular changes. The senescent tissues, organs, or individuals showed a less negative TMP, while tissues with high activity displayed a more negative TMP. And this had been verified in a variety of plants and organs. It was worth noting that when subjected to different adversity signal stimuli, the TMP exhibited different response patterns. In addition, TMP could also be used to detect aging events of membranous organelles.

## Results

### A convenient method for detecting transmembrane potential

**Fig. 1:**
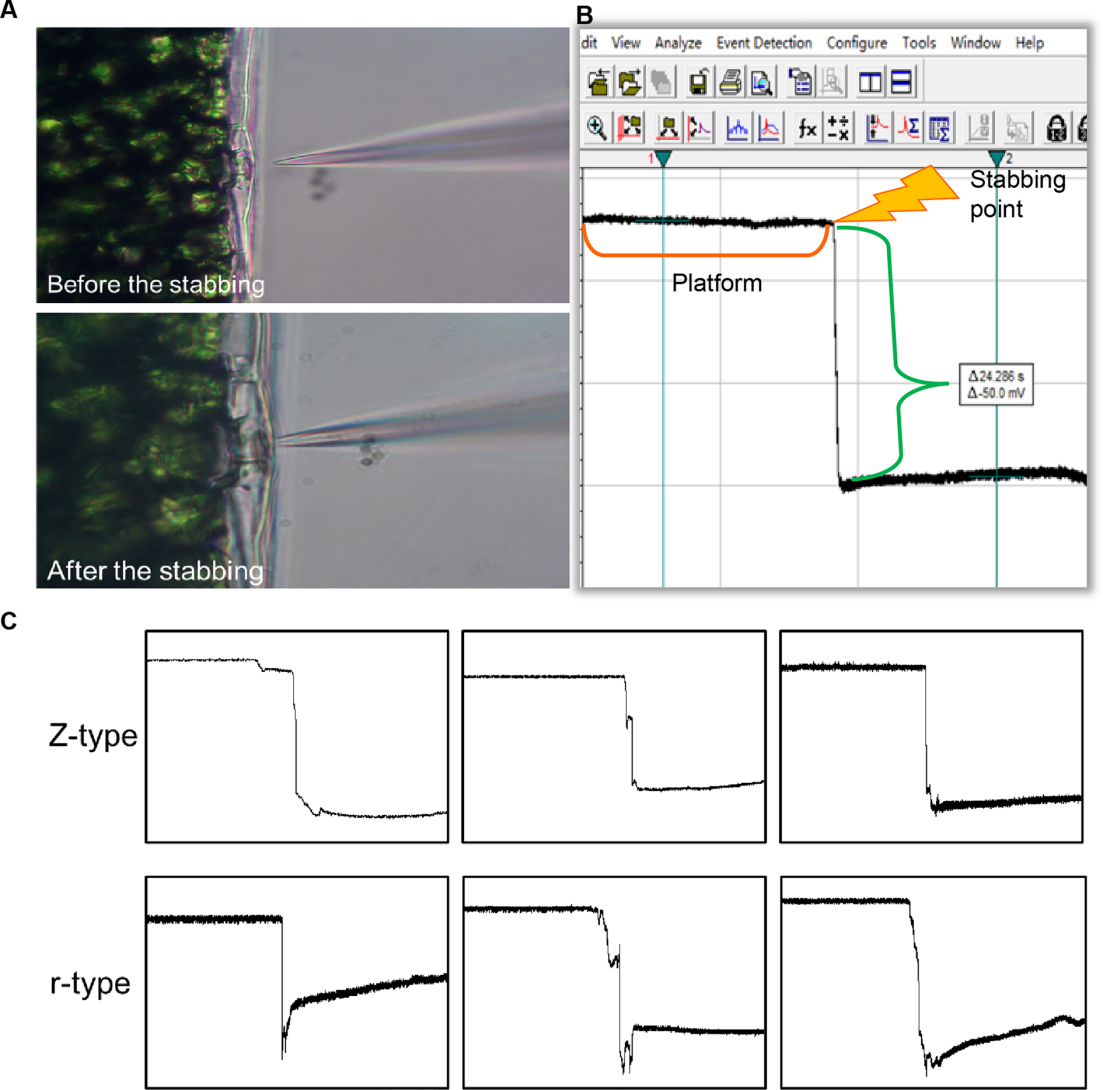
Transmembrane potential (TMP) detection via a patch clamp system. **A** Application of electrodes to pierce *Arabidopsis* mesophyll tissue. **B** Interface of a software Clampfit for measuring the TMP, accurate to 1 decimal. **C** Two common potential diagrams: Z-type and r-type, three represents were showed in each type

We applied a patch-clamp system to monitor the TMP (Additional file 1: Fig. S1). Before detection, the current clamp mode was used with the amplifier set to "I = 0" (Additional file 1: Fig. S2A). The recording of electrical signals used the protocol of gap-free mode, which usually lasted up to 60 s to meet the recording needs (Additional file 1: Fig. S2B). After the electrode was injected into the liquid, the potential would be retained in a relatively stable state. When the electrode was pierced into the plant tissue, the machine captures a significant voltage drop (∆ mV) caused by the potential difference between the inside and outside of the tissue cells (Fig. 1A, B), which represented the TMP of the tissue cells that we monitored. The TMP was captured by a pair of cursors with the software Clampfit 10.6 (Fig. 1B). We proposed the concept of transmembrane potential difference (TMPD), the absolute value of the TMP (|∆ mV|= cusor2−cusor1), which was convenient and easy to describe the difference of TMP mathematically.

Data used for statistics had two typical characteristics, a significant voltage drop and no interference signal. We summarized the common potential diagrams that could be used for statistics into two categories: Z-type and r-type (Fig. 1C). The difference between the two types was mainly due to the variation of the sealing degree after the electrode puncture into plant tissues and cells. Both were common cases and were suitable for data analysis. In addition, a small number of unusable cases showed error signals (Error1–3) that were unusable for statistics (Additional file 1: Fig. S3). We will discuss these cases in detail later.

### Transmembrane potentials differ across life stages in *Arabidopsis*

**Fig. 2:**
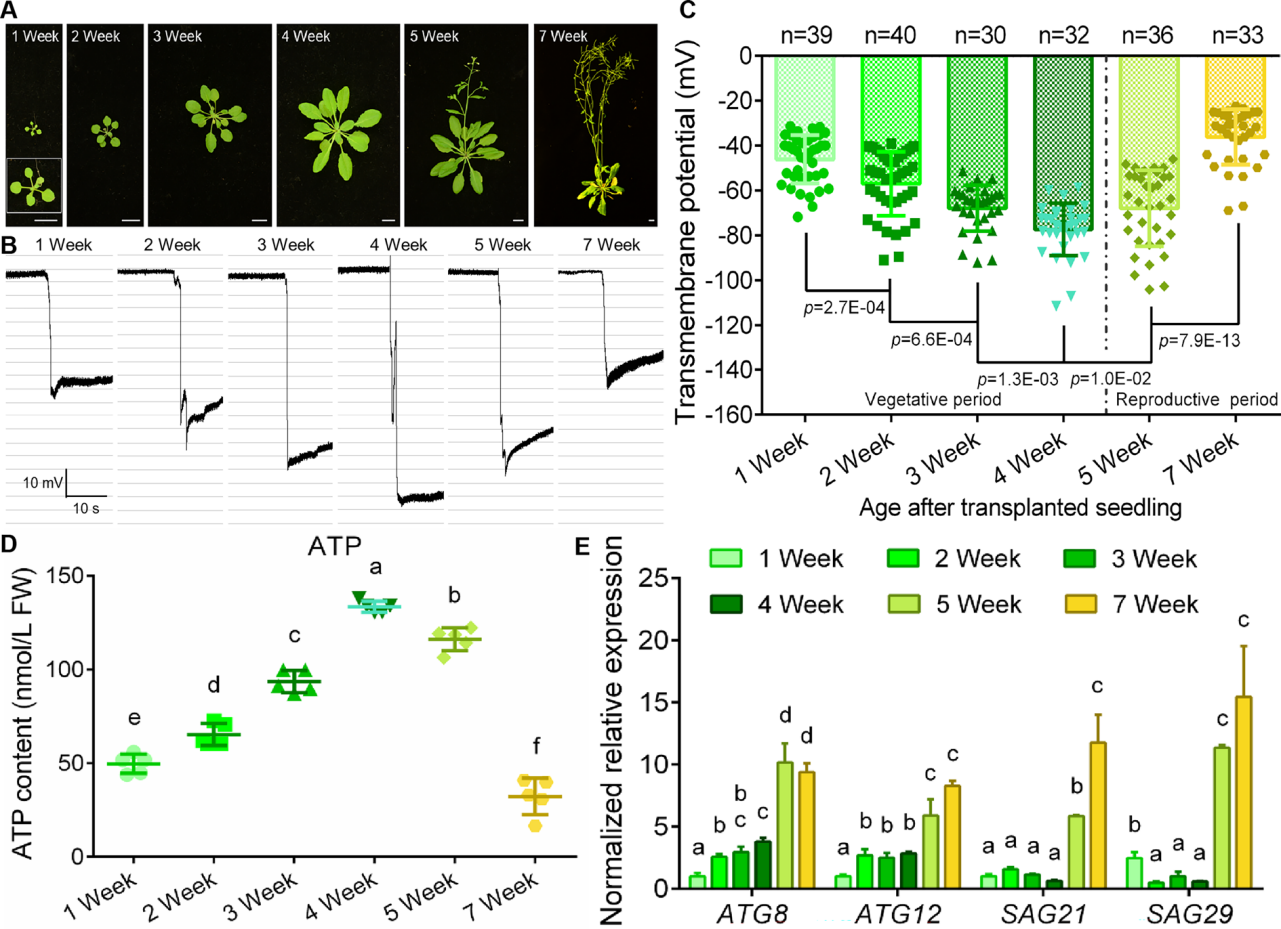
TMP detection in each life stage of *Arabidopsis*. **A** Photographs of growth status of *Arabidopsis* in various life stages, bar = 1 cm. **B** Monitoring diagrams of TMP of rosette leaves in various life stages of *Arabidopsis*, horizontal bar = 10 s, vertical bar = 10 mV. **C** Statistical analysis on TMP of *Arabidopsis* rosette leaves in various life stages. Error bars represent ± S.D., determined by one-way *ANOVA.* **D** Endogenous ATP contents of *Arabidopsis* at various life stages. Error bars represent ± S.D., determined by one-way *ANOVA.* **E** The relative expression levels of *ATG8*, *ATG12* and *SAG*s genes at various life stages of *Arabidopsis.* Error bars represent ± S.E

In order to explore whether the TMP could be invoked as a monitoring index for the condition of plant tissues and cells, we detected the TMP of *Arabidopsis thaliana* rosette leaves from different growth stages (Fig. 2A). The results showed that the TMP of *Arabidopsis* displayed completely different trends at vegetative and reproductive growth stages (Fig. 2B, C). During the vegetative growth period, the TMP of the mesophyll gradually became more negative (hyperpolarization) along with the growth of leaves; while in the reproductive growth period, the TMP of the mesophyll gradually turned less negative (depolarization) with the senescence of leaves (Fig. 2B, C). Near flowering, the TMPD of rosette leaves reached the maximum value (Fig. 2B, C). To dissect whether the changes of TMP have a correlation with the tissue cellular activity, we then measured the endogenous contents of energy molecules at different growth stages of plants. The contents of endogenous ATP, ADP and AMP was determined by high-performance liquid chromatography (HPLC), which can directly reflect the biological activities of plant tissues and cells. The ATP content increased during vegetative growth phase from 1 to 4 weeks and reached a maximum at 4 weeks, then decreased in the following reproductive phase (5 weeks and 7 weeks) (Fig. 2D). The contents of ADP and AMP can be found with a similar trend to that of ATP (Additional file 1: Fig. S4). The expression levels of *ATGs* and *SAGs*, the indicators of senescence genes [33–35], also showed stage differences, with significantly higher expression in the reproductive phase than in the vegetative growth phase (Fig. 2E). This illustrated that the TMP could indicate the senescence of mesophyll cells during plant development process, and the great possibility of using TMP as an indicator to report cell status in plants.

### Transmembrane potential varies among tissues and organs

**Fig. 3:**
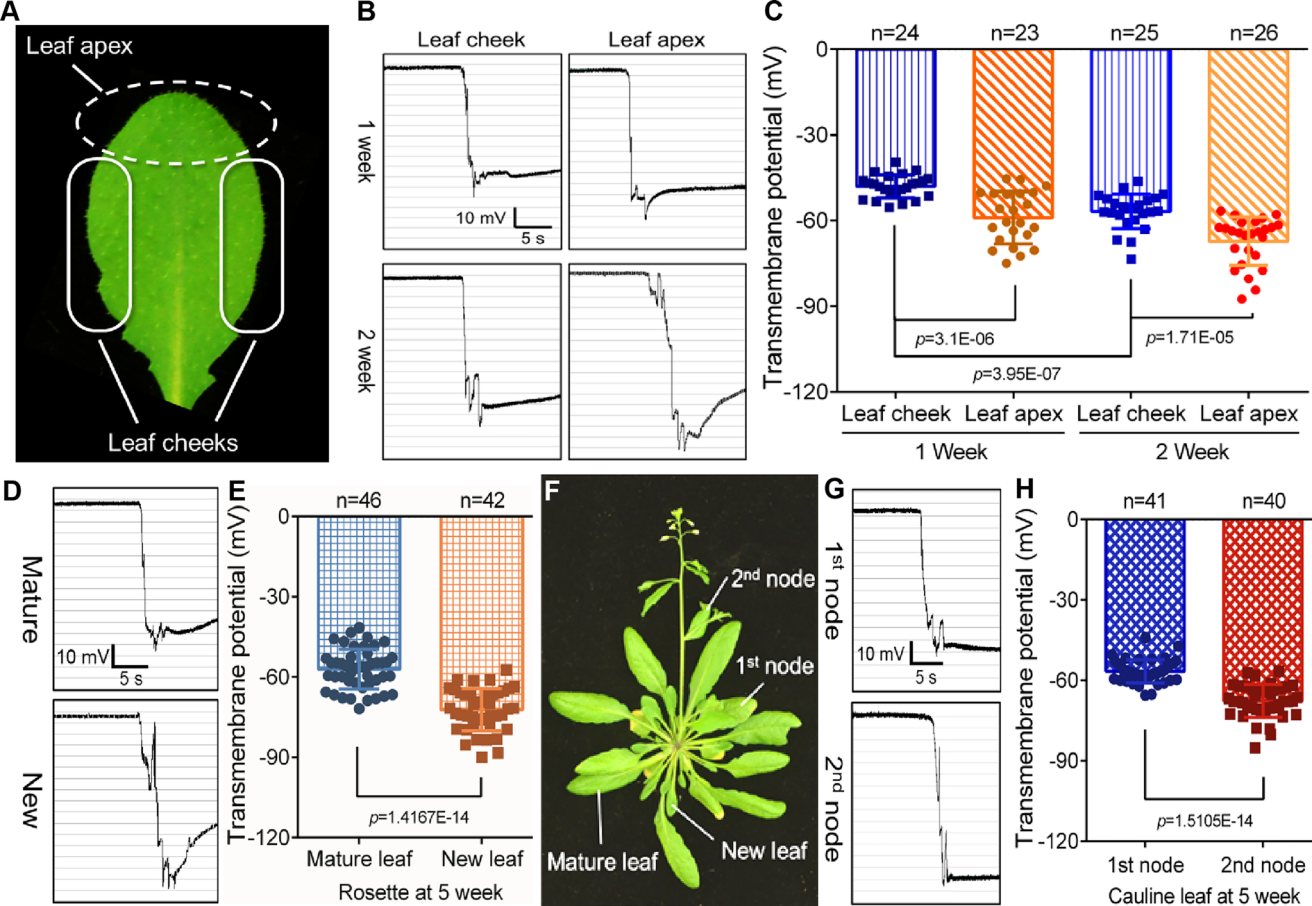
TMP detection in separate tissues of *Arabidopsis.* **A** Photographs of *Arabidopsis* rosette leaf, dotted area = leaf apex, solid line area = leaf cheeks. **B** Monitoring diagrams of TMP in apex and cheek areas of rosette leaves in the 1st and 2nd week, horizontal bar = 5 s, vertical bar = 10 mV. **C** Statistical analysis of TMP in apex and cheek areas of rosette leaves in the 1st and 2nd week. Error bars represent ± S.D., determined by one-way *ANOVA.* **D** Monitoring diagrams of TMP of mature and new rosette leaves in the 5th week, horizontal bar = 5 s, vertical bar = 10 mV. **E** Statistical analysis of TMP of mature and new rosette leaves in the 5th week. Error bars represent ± S.D., determined by one-way *ANOVA.* **F** Photographs of the whole *Arabidopsis* in the 5th week. **G** Monitoring diagrams of TMP of the 1st and 2nd cauline leaves in the 5th week, horizontal bar = 5 s, vertical bar = 10 mV. **H** Statistical analysis of TMP of the 1st and 2nd cauline leaves in the 5th week. Error bars represent ± S.D., determined by one-way *ANOVA*

The TMP of *Arabidopsis* leaves varied at different growth stages. Whether there existed a difference in the TMP of different tissues and organs at the same stage? To understand clearly, we specifically detected the apex and cheeks (lateral margin) of rosette leaves at the same and also different growth stages, separately. The results showed that the TMPs of the two regions were different, and the TMPD in the apex was significantly larger than that in the cheeks (Fig. 3A–C). To further check the generality of the differences in the TMPs among tissues and organs, we also examined the TMPs of mature rosette leaves and new ones. The results showed that the TMP of young leaves exhibited a larger potential difference (Fig. 3D–F). Quantitative RT-PCR analysis indicated that the expression levels of *ATG12a*, *SAG21* and *SAG29* genes were higher in mature rosette leaves than in the young ones (Additional file 1: Fig. S5A). Via detection of TMP in cauline leaves, it was shown that the TMPD of the first cauline leaf was substantially smaller than that of the second cauline leaf in fifth week (Fig. 3F–H). The qRT-PCR results confirmed that the expression levels of the senescence genes (*ATG12a*, *SAG21* and *SAG29*) were higher in the earlier cauline leaves, which coincided with TMP data (Additional file 1: Fig. S5B). These results demonstrated that the tissues and organs of *Arabidopsis* in different growth stages displayed different TMPDs, and new tissues and organs usually exhibited larger TMPDs than mature ones.

### Transmembrane potential indicates senescence of tissue and cells in a variety of plants

**Fig. 4:**
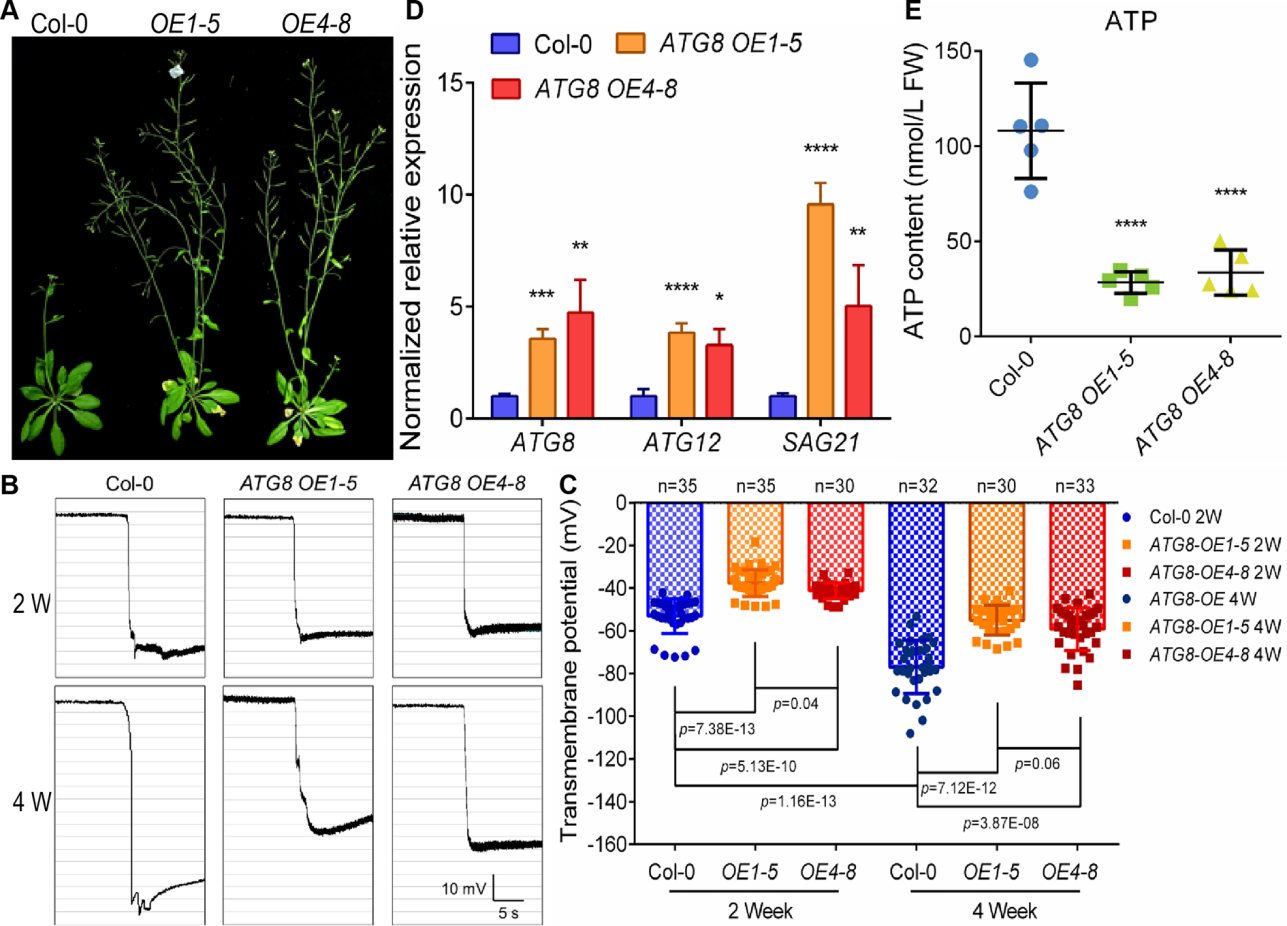
TMP monitoring maps senescence in *Arabidopsis.* **A** Photographs of Col-0 and ATG8-overexpression line in 5th week. **B** Monitoring diagrams of rosette leaf TMPs in 2- and 4-week-old Col-0 and ATG8-OEs, horizontal bar = 5 s, vertical bar = 10 mV. **C** Statistical analysis of rosette leaf TMPs in 2- and 4-week-old Col-0 and ATG8-OE. Error bars represent ± S.D., determined by one-way *ANOVA.* **D** *ATG8*, *ATG12* and *SAG21* expression levels in rosette leaves in 4-week-old *Arabidopsis* Col-0 and ATG8-OEs. Error bars represent ± S.E*.* **E** Endogenous ATP content of *Arabidopsis* rosette leaf in Col-0 and ATG8-OEs. Error bars represent ± S.D., determined by one-way *ANOVA*

According to the electrophysiological test of isolated animal cells, the resting potential of the aging hypocretin neurons was significantly depolarized compared with the younger group [8]. We also observed that during the process of plant senescence, the TMPD of leaf tissue cells decreased gradually (Fig. 2), implying a high possibility that TMP could be exploited to monitor plant senescence. To further explore this hypothesis, we detected and analyzed leaf TMP of *Arabidopsis* Col-0 and *ATG8-OE* at 2- and 4-week-old stages. ATG8 is a core protein in the process of autophagy, which mainly mediates the biogenesis of autophagosomes [36, 37], and its overexpression can cause premature senescence of plants (Fig. 4A). Likewise, for the *ATG8-*overexpressing lines, both 2- and 4-week-old seedlings exhibited significantly lower TMPDs than Col-0 (Fig. 4B, C). The expression levels of the classic senescence genes, *ATG8*, *ATG12* and *SAG21*, were considerably higher in *ATG8-OEs* than that in controls (Fig. 4D). Similarly, the levels of energy substances ATP, ADP, and AMP were all remarkably lower in *ATG8-OEs* (Fig. 4E; Additional file 1: Fig. S6). This phenomenally demonstrated that the value of TMP could reflect the occurrence of plant senescence.

**Fig. 5:**
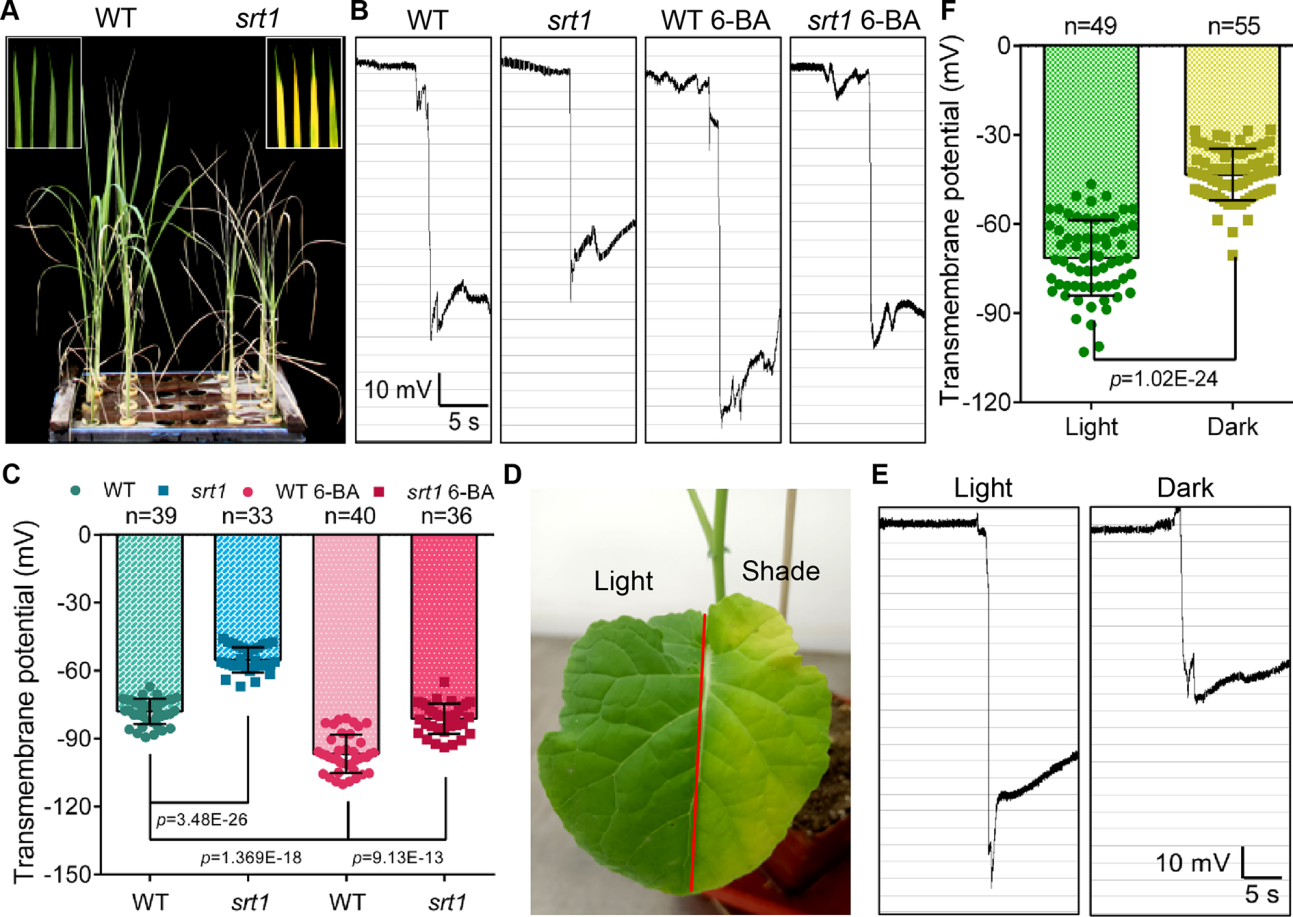
TMP monitoring maps senescence in the crop plants*.* **A** Photographs of rice WT (ZH11) and *srt1*, with magnified figures of leaves in the inset frame. **B** Monitoring diagrams of TMPs in the controls and with 6-BA (5 mg/L) treatments of WT (ZH11) and *srt1* first-leaf at 1-week. TMP was measured after 6-BA treatment for 30 min. Horizontal bar = 5 s, vertical bar = 10 mV. **C** Statistical analysis of TMPs in WT (ZH11) and *srt1* first-leaf in 1-week. Error bars represent ± S.D., determined by one-way *ANOVA.* **D** Photograph of a *Brassica napus* leaf after 10 days of normal light and shade. **E** Monitoring diagrams of TMPs in a light/dark-treated leaf, horizontal bar = 5 s, vertical bar = 10 mV. **F** Statistical analysis of TMPs in light- and dark-treated leaves. Error bars represent ± S.D., determined by one-way *ANOVA*

In addition, to determine whether this detection method had a general application in crop plants, we further identified the TMPs in rice and rape, respectively. Deletion of *OsSRT1*, a silent information regulator 2-related histone deacetylase gene, caused reactive oxygen species (ROS) accumulation and programmed cell death (PCD) initiation in rice, resulting in premature senescence and reduced yield in rice (Fig. 5A) [38, 39]. To avoid the interference of wax, we detected the TMP of rice shoot at 1st week after germination, and the results showed that the tissue TMPD of *srt1* was significantly lower than that of wild-type (WT, ZH11) (Fig. 5B, C), which indicated that the TMP could reflect the premature senescence phenotype of *srt1*. Cytokinin, a development-promoting plant hormone, had been widely reported to delay senescence in plants [40, 41]. After adding 6-BA to the extracellular fluid of the rice shoot, the TMPD of WT was significantly increased, and the decrease of TMPD caused by premature aging in *srt1* was also dramatically recovered (Fig. 5B, C). It was ascertained that TMP could effectively monitor the life condition of rice tissues and cells. Moreover, we simulated the occurrence of leaf senescence in *Brassica napus* by shading for 10 days (Fig. 5D), and then detected the tissue TMP in the normal light area and the shaded area of the same leaf. Although the waxiness of mature leaves interfered with the absolute value of the TMP, the relative potential difference was significant, that is, the TMPD in shaded tissue was significantly lower than that in the normally illuminated tissue (Fig. 5E, F). These results indicated that it was feasible to monitor plant senescence occurrence by measurement of the TMP.

### Transmembrane potential reveals different response patterns in plants under multiple stresses

**Fig. 6:**
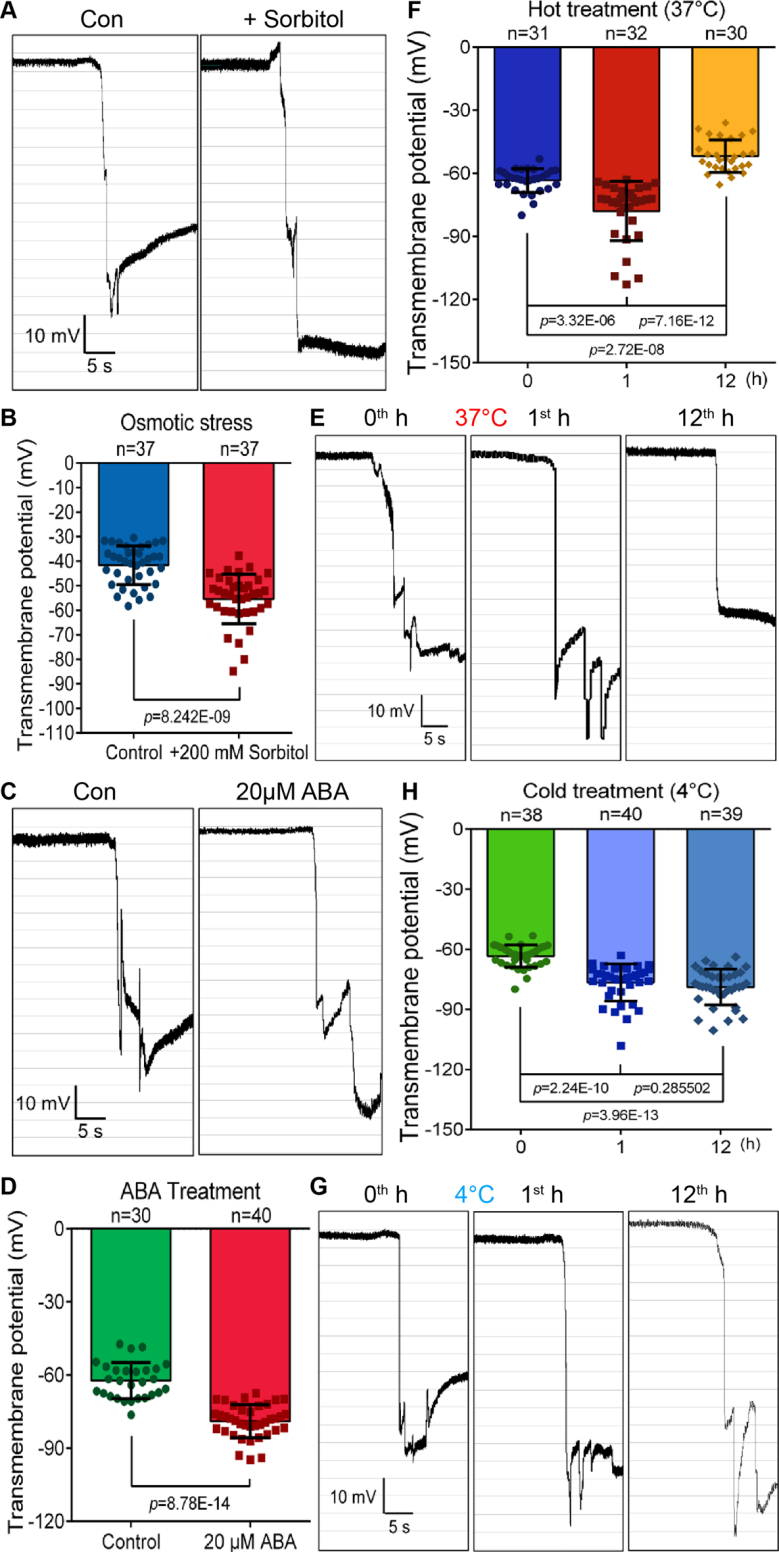
Changes of TMP of plants under different stresses. **A** Monitoring diagrams of leaf TMP in bath solution and high osmotic solution with 200 mM sorbitol (treatment for 30 min), horizontal bar = 5 s, vertical bar = 10 mV. **B** Statistical analysis on TMP of *Arabidopsis* rosette leaves in bath solution and high osmotic solution with 200 mM sorbitol. Error bars represent ± S.D., determined by one-way *ANOVA.* **C** Monitoring diagrams of leaf TMP in control and with treatment of 20 μM ABA for 30 min. Horizontal bar = 5 s, vertical bar = 10 mV. **D** Statistical analysis on leaf TMP of *Arabidopsis* rosette in control and with 20 μM ABA. Error bars represent ± S.D., determined by one-way *ANOVA.* **E** and **G** Monitoring diagrams of leaf TMP before treatment (0th h), with 1 h simulate heat (37 °C, F)/cold (4 °C, H) stress (1st h), and after recovering for 11 h (12th h). Horizontal bar = 5 s, vertical bar = 10 mV. **F** and **H** Statistical analysis on leaf TMP before (0th h), with 1 h simulate heat (37 °C, F)/cold (4 °C, H) stress (1st h), and recover 11 h (12th h). Error bars represent ± S.D., determined by one-way *ANOVA*

Apart from biological senescence, plants were also experienced premature senescence after exposure to various stresses. We then further investigated whether the senescence signal of tissues and cells in response to multiple stresses could be identified via the measurement of the TMP in the tissues and cells. According to previous studies, osmotic stress can induce premature senescence of plant leaves, which was associated with the induction of high expression of *SAGs* in plants [42, 43]. After addition of 200 mosm/kg sorbitol to the bath solution for 30 min, the TMPD of leaf tissue did not decrease significantly, but increased unexpectedly (Fig. 6A, B). It was speculated that the hypertonic solution might promote a large amount of efflux of intracellular H_2_O, which led to the increase of leaf TMPD [3, 44]. Since plant hormone abscisic acid (ABA) has been widely reported to be involved in the induction of plant senescence [17, 45–47], we added ABA to the bath solution to observe its effect on TMP. However, the results unexpectedly showed that exogenous addition of 20 μM ABA promoted the TMPD (Fig. 6C, D), which will be discussed later.

To further clarify whether other stresses also led to hyperpolarization of plant tissue, we examined the changes of TMP in response to cold and heat stresses. The results showed that the TMPs of *Arabidopsis* leaves were hyperpolarized after 1 h of cold and heat stress treatments (Fig. 6E–H). Different stress treatments leading to the hyperpolarization of tissue TMP seems to be a common phenomenon, but it contradicted the phenomenon that aging presents a decrease in TMPD (Fig. 4). Referring to previous reports, we found that exogenous stress stimuli induce a stress response in cells, which leads to hyperpolarization of the cell membrane potential [48], but such a stress response is typically a short-term effect that does not last for long. Therefore, we treated the *Arabidopsis* at the extreme temperature for 1 h and took them back to a normal growth environment for 11 h, and then further analyzed their TMP. The results showed that after 11 h of recovery, the TMPD of leaf treated with heat stress was significantly lower than that before treatment (Fig. 6E, F). This indicated that the TMP hyperpolarization occurred quickly in plant tissue cells after being pretreated at 37 °C for 1 h (Fig. 6E, F). After that, the TMPD was reduced due to aging events. Differently, the tissue leaf TMP showed a tendency of hyperpolarization after 1 h of cold treatment (4 °C). Even though the leaf recovered for 11 h in suitable conditions, it still showed a higher value compared with the control group (0th h) (Fig. 6G, H). This showed that the mechanisms of plants in response to cold stress and heat stress might be different, and *Arabidopsis* probably had a stronger tolerance to cold stress than the heat stress.

### Transmembrane potential can monitor the decline of membranous organelles

**Fig. 7:**
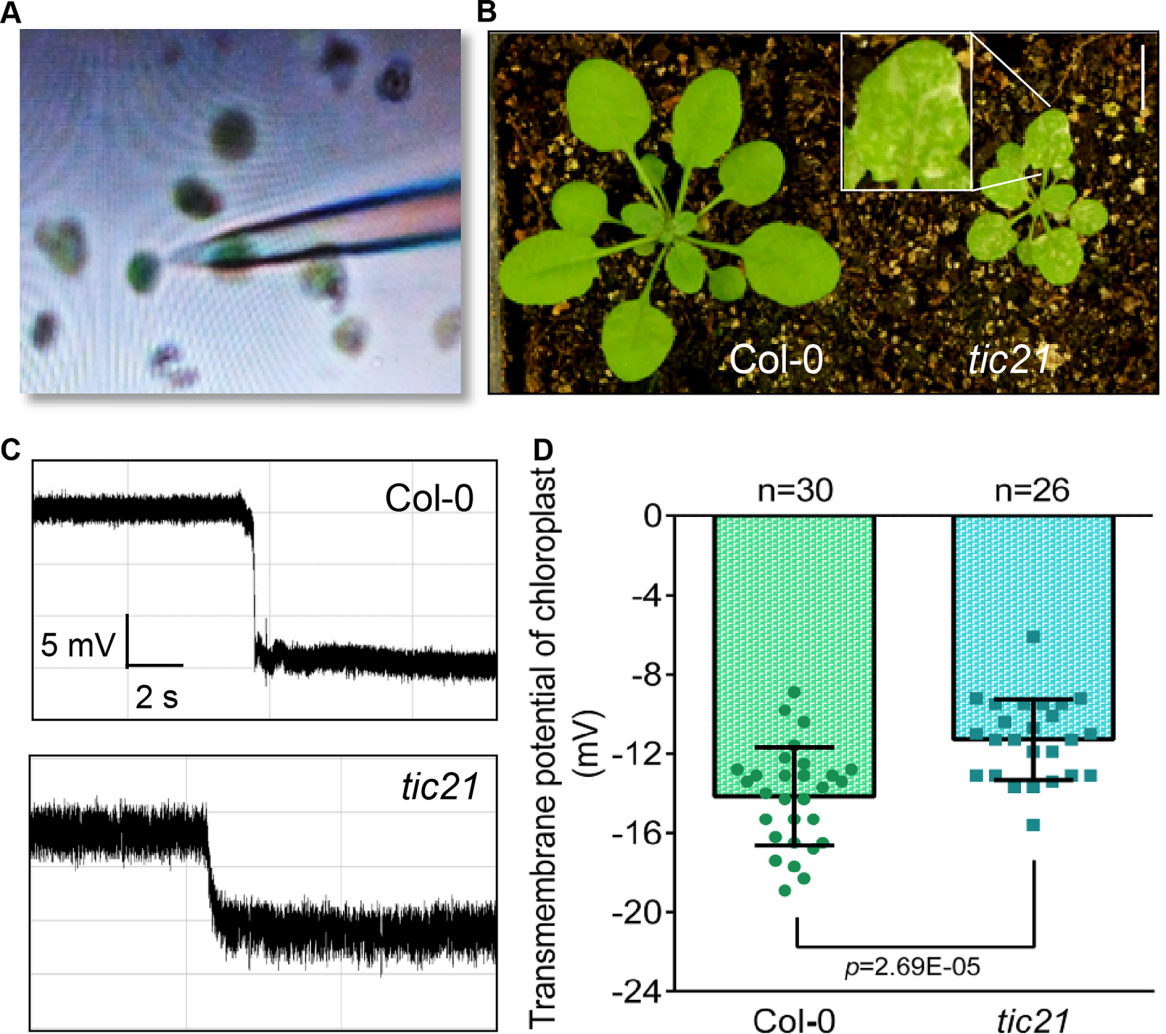
Detection of organelle TMP. **A** Photograph of using electrodes to pierce *Arabidopsis* chloroplasts, the diameter of the electrode tip = 1–1.5 μm. **B** Photographs of *Arabidopsis* Col-0 and *tic21* seedlings. White bar = 1 cm. **C** Monitoring diagrams of TMP of chloroplasts. Horizontal bar = 2 s, vertical bar = 5 mV. **D** Statistical analysis on chloroplast TMP. Error bars represent ± S.D., determined by one-way *ANOVA*

As illustrated above, we used this handy TMP assay to reveal the possibility of monitoring of cellular activity in plant tissues. We wanted to explore whether such a hallmark could be used in a broader study of cell physiology, since patch-clamp technique for detecting ion channels in organelles has long been reported [49–51]. There also have been attempts to detect plant chloroplast TMP using two electrodes method [52], so we attempted to use one electrode to monitor the TMP of chloroplasts (Fig. 7A). Deletion of *Tic21*, encoding part of the inner membrane protein transduction pathway of chloroplasts, can lead to defective chloroplast function and whitening of leaves [53, 54] (Fig. 7B). We isolated chloroplasts from Col-0 and *tic21* lines and monitored their TMP utilizing the same system as described above (Fig. 7A, B). Since the chloroplast is a free individual and cannot be pierced (Fig. 7A), we utilized a sharp increase of negative pressure to penetrate the chloroplast membrane. A sharp drop in voltage was recorded, which indicated the TMP of the chloroplast (Fig. 7C). The TMPD of *tic21* chloroplasts was significantly lower than that of Col-0, indicating the low chloroplast activity in *tic21*. This suggested that the activity of chloroplasts could also be monitored via the TMP (Fig. 7C, D). This also revealed that TMP can be used to monitor the activity of membranous organelles at subcellular level.

## Discussion

### Details for normalized plant tissue membrane potential recording

The advantage of this method is that the range of tissue membrane potential can be monitored by a simple puncture [29, 31, 32, 55–57]. The operation is extremely simple than the whole-cell recording by patch-clamp, and the obtained data are much close to the native environment than that in the isolated protoplasts (Fig. 1A). The native environment of plant tissues may bring complex interference, and individual differences of samples are also important interference factors in data statistics. According to previous work and experiences, we found that the statistical results of the membrane potential of all samples generally showed a normal distribution [29]. In order to make the results more accurate, we suggest that the number of recorded samples preferably exceeds 30.

According to the analysis of a large number of membrane potential data, we divide them into two types: Z-type and r-type (Fig. 1C). The electrode tip after piercing forms an excellent seal with the tissue cell membrane (Additional file 1: Fig. S3D-a), and the potential after the piercing is maintained at a stable level (Fig. 1C). Incidentally, such states can be used for feedback observations of some additional stimuli. On the other hand, r-type implies a transient poor sealing after puncture, but the membrane potential tends to be stable (Fig. 1C). After a negligible transient incomplete sealing, as shown in Additional file 1: Fig. S3D-b, which may be caused by a tiny gap, the electrodes then adhere to the membrane and tend to recover sealing. The two types data are both suitable for statistical analysis. Except for the two cases, a small number of error data are improper for statistics, the common problems are charted in records Error1-3 (Additional file 1: Fig. S3A–C). An experiment details are given below to help researchers avoiding unnecessary bugs. In the case of “Error1”, an extra "Peak1" signal before "Valley1" was recorded owing to the electrode has not been penetrated into the tissue cell quickly (Additional file 1: Fig. S3A). Operation deftly will be helpful to effectively avoid such a novice problem. Another case, Error2, appears a small potential oscillation and keeps for seconds after stabbing (Additional file 1: Fig. S3B). That may due to poor puncture, no sealing has been formed even momentarily. Error3, a relatively rare error, is caused by the breakage or blockage of the electrode tip. Timely replacement of the glass electrode and the bath fluid helps to avoid such terrible situations.

### Transmembrane potential can monitor plant tissue condition

Cell death is a crucially biological process for the vast majority of life [5]. Disrupting intracellular homeostasis, either by excessive or scanty cell death, is a mark of typical pathological and physiological pathways [5]. In humans and animals, the stability of cell and organelle membranes is the basis for maintaining normal cellular activity, and the destruction of the membrane system will directly induce cell senescence or apoptosis [5, 58]. Ion channel activities and depolarized membrane potential are linked with oncogene-induced senescence [59]. In plants, senescent plant tissues and organs also have electrolyte leakage and membrane potential imbalance [60], especially when suffering from toxic metal stress and apoptosis [11, 61–64]. In our work, by tracking the magnitude of the leaf TMP during the entire growth cycle of *Arabidopsis*, we found that the TMP of *Arabidopsis* leaves showed opposite trends at different growth stages (Fig. 2). During the reproductive growth phase, with the senescence of leaves, the TMP showed depolarization, that is, the TMPD moderately reduced (Fig. 2). However, in the vegetative growth phase, the TMP of leaves showed a tendency of hyperpolarization, that is, the leaf TMPD was positively correlated with the leaf age (Fig. 2). Especially, when we examined the leaves of different regions of 5-week-old *Arabidopsis*, we found that the TMP of new rosettes was significantly higher than that of the mature leaves (Fig. 3D–F), and the TMPD of the first cauline leaves was also significantly lower than that of the second one (Fig. 3F–H). These results further suggested that the overall senescence event of plants is initiated and progressed in reproductive growth phase. This points out that when measuring the TMP to reflect the senescence degree of a particular strain, the plant tissues and organs at the vegetative growth stage should be selected first, and at least the uniformity of the stage should be retained. In detection of *Arabidopsis* progeria line *ATG8-OE*, both the 2- and 4-week-old leaves showed lower TMPD compared with the WT (Fig. 4), which is in line with the above view.

During detection of TMP of the leaves, we unexpectedly found that the apex and lateral margin of the same leaf showed different TMP. The apex region showed a larger TMPD (Fig. 3A–C), which indicated that the leaf apex region probably has a higher cellular activity. This interesting phenomenon is consistent with previous study that the apex region of *Arabidopsis* leaves is a key proliferation region during leaf growth [65]. It also suggests that uniformity should be maintained for the location of the detected tissue. Taking the detection of rosettes as an example, the consistency of detection position should be ensured, and the TMP of the leaf lateral margin region remains relatively stable (Fig. 3A).

In our previous work, it was found that the TMPD of the fruit pulp cells of the orange variety "*Gannan No.1*" with good freshness was significantly higher than that of the conventional variety "*Newhall*" at 45 d and 135 d after harvest [66]. We have verified it in crops of rice and *Brassica napus* in the present study. The results showed that the leaf TMPD of the progeria line *srt1* is significantly lower than that of WT, and exogenous application of 6-BA can significantly increase the leaf TMPD. In *Brassica napus*, the TMPD of leaves that have been induced into senescence was also lower than that of the controls. These studies prove that the TMP detection method can be universally and feasibly applied in a variety of plants.

### Transmembrane potential responses in different modes upon multiple stresses

In the present study, the shading treatment can induce depolarization of the TMP in *Brassica napus* leaves (Fig. 5D). A number of stresses including salinity [67, 90], osmosis [68], drought [67, 69], toxic metals [70, 71], high temperature [61, 72] and frost [73] can lead to premature senescence and even apoptosis in plants. To observe whether the TMP of plant tissues would be depolarized and report the earlier senescence signal after stress stimuli, in contrast to the expectations, the TMP of *Arabidopsis* rosette leaves under hypertonic environment was hyperpolarized instead of depolarized (Fig. 6A, B). We speculated that this may be caused by the efflux of the H_2_O and accumulation of various intracellular ions, resulting to hyperpolarize the TMP [22, 74]. After treating the leaves with the senescence-promoting phytohormone ABA, we were surprised to find that the TMP of leaves was also hyperpolarized, but not depolarized (Fig. 6C, D). The effects of 1 h treatment of high and low temperatures on *Arabidopsis* rosette leaves exhibited the same result of hyperpolarized TMP (Fig. 6F–H).

Stress responses are triggered by the stresses that the cells suffered. [24, 75, 76]. The occurrence of stress response affects a series of cellular activities, and the overall performance is the hyperpolarization of TMP [2, 48]. This phenomenon is often a short-term effect, which is probably related to the instantaneous oscillation of intracellular Ca^2+^ and K^+^ balance crossing the cell membrane [24, 76–79]. In barley, ABA induced hyperpolarization of aleurone protoplasts membrane potential and reached the maximum after 20 min of treatment [80]. In root epidermal cells of *Arabidopsis*, ABA induced membrane hyperpolarization, which is due to K^+^ efflux through the GORK channel [81]. In guard cells, in addition to activating the GORK channel, ABA inhibits inward K^+^ channels and promotes K^+^ efflux [82]. These studies indicated that after a short time (tens of minutes) of ABA treatment, the TMP of plant cells will be hyperpolarized, establishing ion concentration gradients across the cell membrane. Compared to the senescence induced by light-proof treatment for a long time (10 days), the interval between the stress treatment and detection seems too short (1 h) to detect the signal of senescence. As for the heat and cold stress analyses, after a short time (1 h) stress treatments and further a half-day recovery in a suitable growth environment, the TMP of rosette leaves was depolarized under the high-temperature stress treatment (Fig. 6E, F), but was kept in a hyperpolarized state under the low temperature treatment (Fig. 6G, H). This indicates that *Arabidopsis* has a more sustained tolerance to low temperature, and probably has different defense mechanisms in response to cold and heat stresses. The present study reveals that the maintenance of TMP hyperpolarization is an index of plant resistance ability to environmental stresses.

### Perspective application of transmembrane potential detection in membranous organelles.

The intracellular membranous organelles have TMP causing by differential distribution of electrolytes in the cytosol and lumina. Among them, mitochondrial TMP has been reported mostly. The steady-state of the mitochondrial TMP is closely linked to protein activity on the double-layer membrane [5]. The mitochondrial TMP is an important reference parameter for apoptosis in animal and plant cells [83–85]. However, mitochondria are too small to be easily detected by the present method. Chloroplast, a large, numerous and full-shaped organelle, is feasible for the simple TMP detection method. For the detection of *Arabidopsis* chloroplasts, it is important to separate and purify clean chloroplasts. Due to the separation of organelles, in the experimental operation, it is necessary to first adsorb a chloroplast by a mild negative pressure, and then immediately give a strong negative pressure to suck and impale it (Fig. 7A). Upon piercing into the chloroplast, the system will record a negative voltage drop, which indicates the chloroplast TMP (Fig. 7D). Meanwhile, the measurement results of the chloroplast development-deficient mutant *tic21* confirmed the feasibility of the detection of chloroplast TMP (Fig. 7B-D). In addition, the vacuole, the largest organelle in plant cells, is also crucial for the homeostasis of its TMP [86, 87]. The method described above also has the potential to facilitate better understanding of tonoplast TMP.

## Conclusions

Here we applied a convenient TMP detection method that can report the senescence signal of plant tissues and cells in vivo. It can also indicate the potential of plant tolerance to environmental stress, which would be suitable for the selection and breeding of crop cultivars with high resistances/tolerances to multiple stresses.

## Materials and methods

### Plant materials and growth conditions

The *Arabidopsis thaliana* ecotype Col-0 served as the WT control. The transgenic *ATG8-OE* lines were made using 35S promoter, containing YFP fluorescent tag, and identified by fluorescence intensity and RT-qPCR. The *tic21* mutants, obtained using CRISPR/Cas9 method, were kindly provided by Ms. Jumei Zhang from College of Life Science and Technology, Huazhong Agricultural University. The *Oryza sativa* ecotype ZH11, used as the WT control, and *srt1* mutant were kindly provided by Prof. Jie Luo from Tropical Crop College of Hainan University. The *Brassica napus* ecotype Westar seedlings were kindly provided by Dr. Xuan Yao from College of Plant Science and Technology, Huazhong Agricultural University. *Arabidopsis* seeds were germinated on humid soil (peat soil: vermiculite: perlite = 1: 1: 1) for 2 d at 4 °C in darkness for vernalization, and then transferred to a phytotron at 21 °C with a 16-h light and 8-h dark photoperiod. After growth for 7—10 days, the four-leaf stage seedlings were transplanted into separate pots. Rice seeds were surface-sterilized in 3% (v:v) H_2_O_2_ for 10 min, then rinsed and imbibed for 48 h in aerated distilled water at 30 °C. After a further 5–7 days, germinated seeds were placed in glass tubes with agarose medium, and grew in a greenhouse or a controlled growth chamber.

### Components of electrode solutions

The pipette solution used to detect *Arabidopsis* and *Brassica napus* contains 3 M KCl, and the bath solution contains 5 mM KCl, 1 mM CaCl_2_, 1 mM MgCl_2_, 10 mM MES and sorbitol to 486 mOsm, adjust pH to 5.5 with Tris. The solutions were modified with reference to patch clamp technique and non-invasive microelectrode ion flux measurement (MIFE) [55, 88]. In rice, the pipette solution contains 0.1 M KCl and the bath solution contains 5 mM MES, 0.5 mM CaCl_2_ and 0.05 mM KCl, adjust pH to 6.0 with Tris-HCl [30]. All solutions were sterile filtered (0.22 μm) and stored at 4°C. General chemical reagents were purchased from Sigma (USA) or Aladdin (CHN).

### Stress conditions and hormones treatment

In the stress experiment, the *Arabidopsis* leaves of 3–4 weeks were detected for the first time before treatment, the second time after 1h of cold (4 °C) and heat (37°C) treatment, and the third time after the seedlings returned to 21°C conditions for 11h. ABA (Sigma) was dissolved into 10 mM storage solution with absolute ethanol, and diluted to the working concentration of 20 μM. 6-BA (Yeasen) was dissolved into 5 mM storage solution with 0.1 M NaOH, and diluted to the working concentration of 10 μM. Osmotic solution was obtained with addition of 200 mM sorbitol to the bath solution. All solutions were perfused to the samples via a pump during detection.

### Transmembrane potential detection equipment and method

A patch-clamp system was used to detect cell TMP in plant tissue (Additional file 1: Fig. 1S). The system includes an amplifier (Axopatch 200B, Molecular Devices, USA), a digital—analog converter (Digidata 1550B, Molecular Devices, USA), a micro-manipulator (MP-285, USA) and an inverted microscope (Olympus, Japan). The current-clamp mode was selected as “I = 0” on amplifier (Axopatch 200B, Molecular Devices, USA) and the gap-free protocol mode (software, Clampex10.6) was used to collect data (Additional file 1: Figs. S1, S2). The plant tissue was fixed in a small round dish and immersed in an isotonic bath solution. A glass electrode with pipette solution was mounted on the AgCl electrode, and was then placed into the bath solution close to the sample via a micromanipulator. The aperture of the glass pipette tip was 0.5–1.5 µm, which is similar to the pipette tip used in the whole-cell patch clamp experiment. The glass pipette was pulled with a micropipette puller (Sutter P-1000, Sutter Instrument, USA) and has a longer neck and lower curvature to reduce the possibility of broken. Before the recording, after the potential was maintained at a stable state, the electrode was pierced quickly into the plant tissue. The TMP (∆mV) was captured by a pair of cursors in the voltage recording curves with the software Clampfit10.6. Normally, cursor1 was set in the platform area, cursor2 was set at a relative stable state usually 5–10 s after the stabbing point. For r-type recording graph, the “valley1” (Additional file 1: Fig. S3A) should be circumvented and data of cursor2 were the average amplitudes of potential recording ranged from 5 to 10 s after “valley1”. To measure the TMP of *Arabidopsis* rosette leaves at different growth stages, the lateral margin areas of rosette leaves in sunlight side (adaxial side) were selected and the microelectrode was punctured into the mesophyll cells.

### Isolation and extraction of intact chloroplasts of *Arabidopsis*

3- to 4-week-old *Arabidopsis* leaves (1–3 g) were freshly collected in the morning, then smashed with a blender in pre-cooled chloroplasts isolation buffer (CIB: 0.5 M Sorbitol, 50 mM Tricine, 1 mM DTT, 1 mM EDTA, 1 mM MgCl_2_, 1% BSA). Samples were filtrated with double-layer membrane, and centrifuged at 200 g for 3 min at 4 °C in a 50 mL tube. White sediment (starch and other impurities) appeared below. The green part of the supernatant was moved into a new 50 mL tube, washed with CIB and centrifuged at 1000*g* for 7 min. The sediment was collected and mildly resuspend with 1 mL CIB. 40% precoll (1.5 mL) was added into a new tube, and 0.5 mL resuspension was transferred into the upper layer in the same tube. After centrifugation at 1700*g* for 6 min at 4 °C, the intact chloroplasts were precipitated at the bottom of the tube. Removing the supernatant, the intact chloroplasts were resuspended with 1–2 mL CIB (without BSA) and stored on ice for TMP detection. General chemical reagents were purchased from Sigma (USA) or Aladdin (CHN).

### Quantitative Real-time PCR

Total RNA from plant leaves was extracted using the TriZol method with Total RNA Isolation Reagent (BS258A, Biosharp). Then, it was reverse transcribed into cDNA using Hifair^®^ III 1st Strand cDNA Synthesis SuperMix with gDNA digester plus (11141ES60, YASEN), and the cDNA was used as a template for PCR amplification. Real-time PCRs were performed in a Bio-Rad CFX96 Real-Time System (C1000 Touch™, USA) with 2 × SYBR qPCR Mix (PC3302, Aidlab). The relative gene expression was normalized to the expression of ubiquitin5 (*UBQ5*). The primers used in this study are listed in Additional file 1: Table S1. The mean threshold cycle values for the genes tested in the study were calculated based on three replicates.

### HPLC detection of endogenous ATP, ADP, and AMP contents

The leaves were grounded in liquid nitrogen and dissolved in an appropriate amount of 50% acetonitrile. The samples were ultrasonically crushed for 30 min, and then centrifuged at 15,000*g* for 10 min at 4 °C. The supernatant was filtered (0.22 micron) and collected for analysis. For metabolome analysis, samples were analyzed using a HPLC-based targeted method [89]. The analytical conditions were shown as follows. HPLC: column, shim-pack GISS C18 (pore size 1.9 µm, length 2.1 × 100 mm); solvent system, A, water (0.04% acetic acid), B, acetonitrile (0.04% acetic acid); gradient program, 0 min, 5% B; 12.0 min, 95% B; 13.2 min, 95% B; 13.3 min, 5% B; 15.0 min, 5% B; flow rate, 0.4 mL min^–1^; temperature, 40 °C; injection volume: 2 µL. The targeted metabolic profiling analysis was conducted by using a scheduled multiple reaction monitoring (MRM) via LC-ESI-QQQ-MS/MS system (LCMS-8060, SHIMADZU, Japan). The ESI source operation parameters were listed as follows: nebulizing gas flow, 3 L min^–1^; heating gas flow, 10 L min^–1^; interface temperature, 500 °C; DL temperature, 250 °C; heat block temperature, 400 °C; drying gas flow, 10 L min^–1^. The recorded data were handled with LabSolutions 5.91 software. Standards ATP, ADP and AMP saltsslat were purchased from Aladdin (CHN).

## Supplementary Information

**Additional file 1: Figure S1.** Using patch-clamp system to detect plant tissue TMP. **Figure S2.** TMP detection parameter settings in patch-clamp system. **Figure S3.** Unconventional potential shapes. **Figure S4.** Contents of ADP and AMP at different growth stages. **Figure S5.** Expression of senescence genes in different organs. **Figure S6.** Contents of ADP and AMP in Col-0 and ATG8-OEs. **Table S1.** Primers used for quantitative real-time PCR.

## Abbreviations

ABA: Abscisic acid
TMP: Transmembrane potential
TMPD: Transmembrane potential difference
ATP: Adenosine triphosphate
ADP: Adenosine diphosphate
AMP: Adenosine monophosphate
RNA: Ribonucleic acid
cDNA: Complementary DNA
qRT-PCR: Quantitative reverse transcription–polymerase chain reaction
HPLC: High Performance Liquid Chromatography
