# Supplementary material for: Transmembrane potential, an indicator in situ reporting cellular senescence and stress response in plant tissues
Source: Plant Methods. 2023 Mar 21;19:27. doi: 10.1186/s13007-023-01006-0 (PMC10029184; doi:10.1186/s13007-023-01006-0)
Supplement: Supplementary file 1 — Additional file 1: Figure S1. Using patch-clamp system to detect plant tissue TMP. Figure S2. TMP detection parameter settings in patch-clamp system. Figure S3. Unconventional potential shapes. Figure S4. Contents of ADP and AMP at different growth stages. Figure S5. Expression of senescence genes in different organs. Figure S6. Contents of ADP and AMP in Col-0 and ATG8-OEs. Table S1. Primers used for quantitative real-time PCR. [file 13007_2023_1006_MOESM1_ESM.pdf]

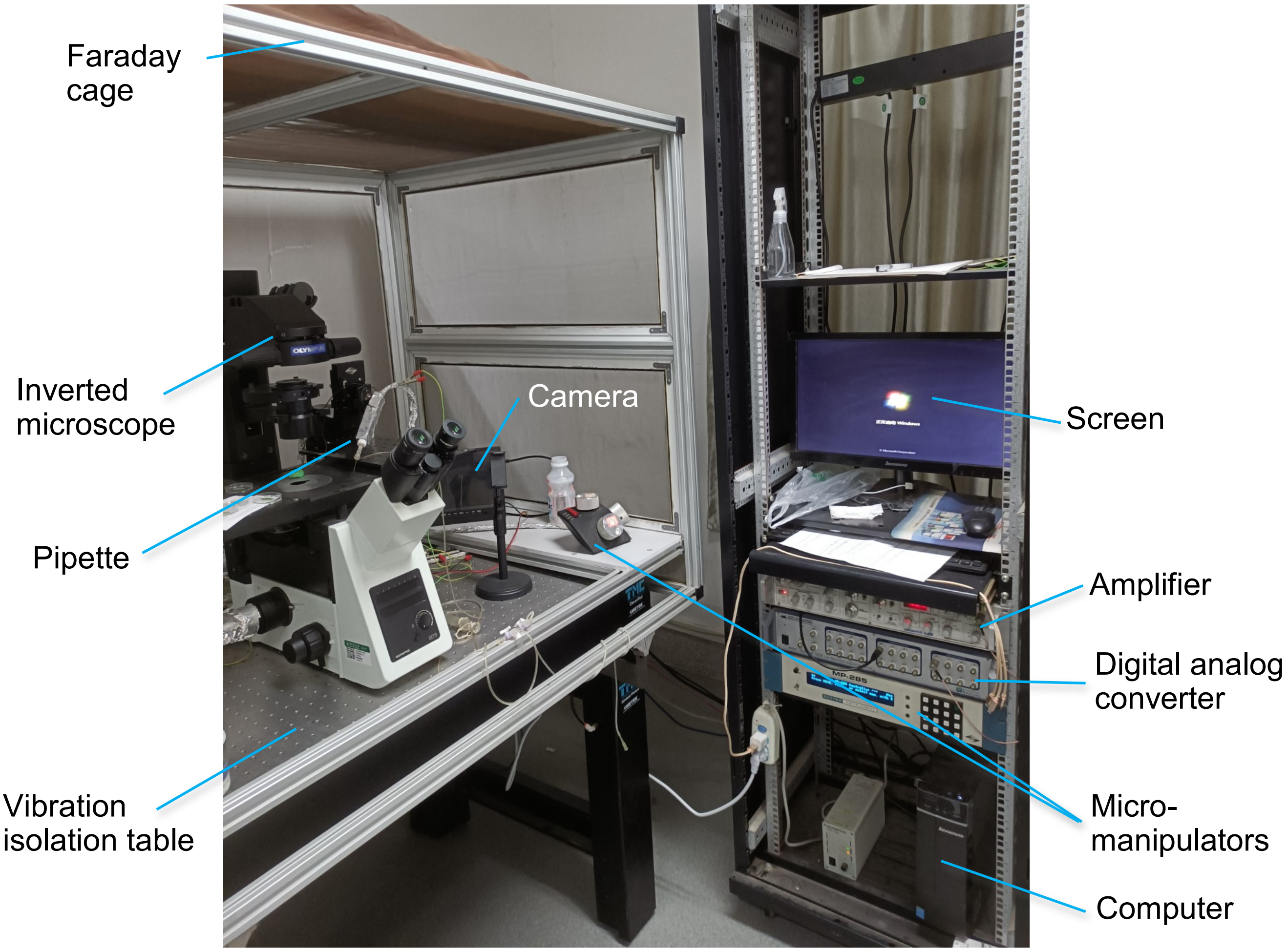

Figure S1. Using patch-clamp system to detect plant tissue TMP.

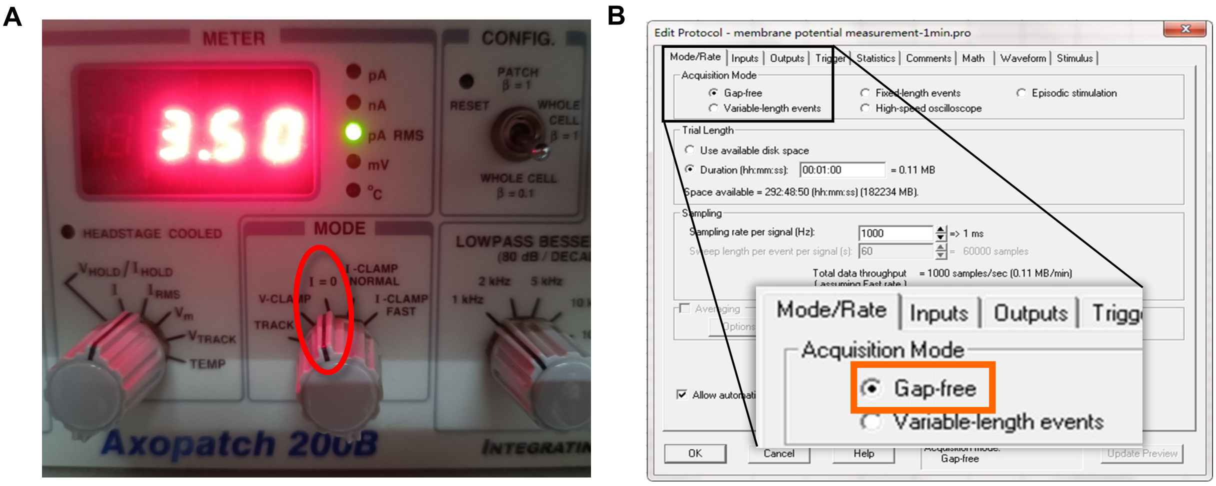

Figure S2. TMP detection parameter settings in patch-clamp system.

A, Axopatch 200B amplifier triggered to mode “I=0”. B, Set acquisition mode of protocol to “gap-free” by software Clampex 10.6.

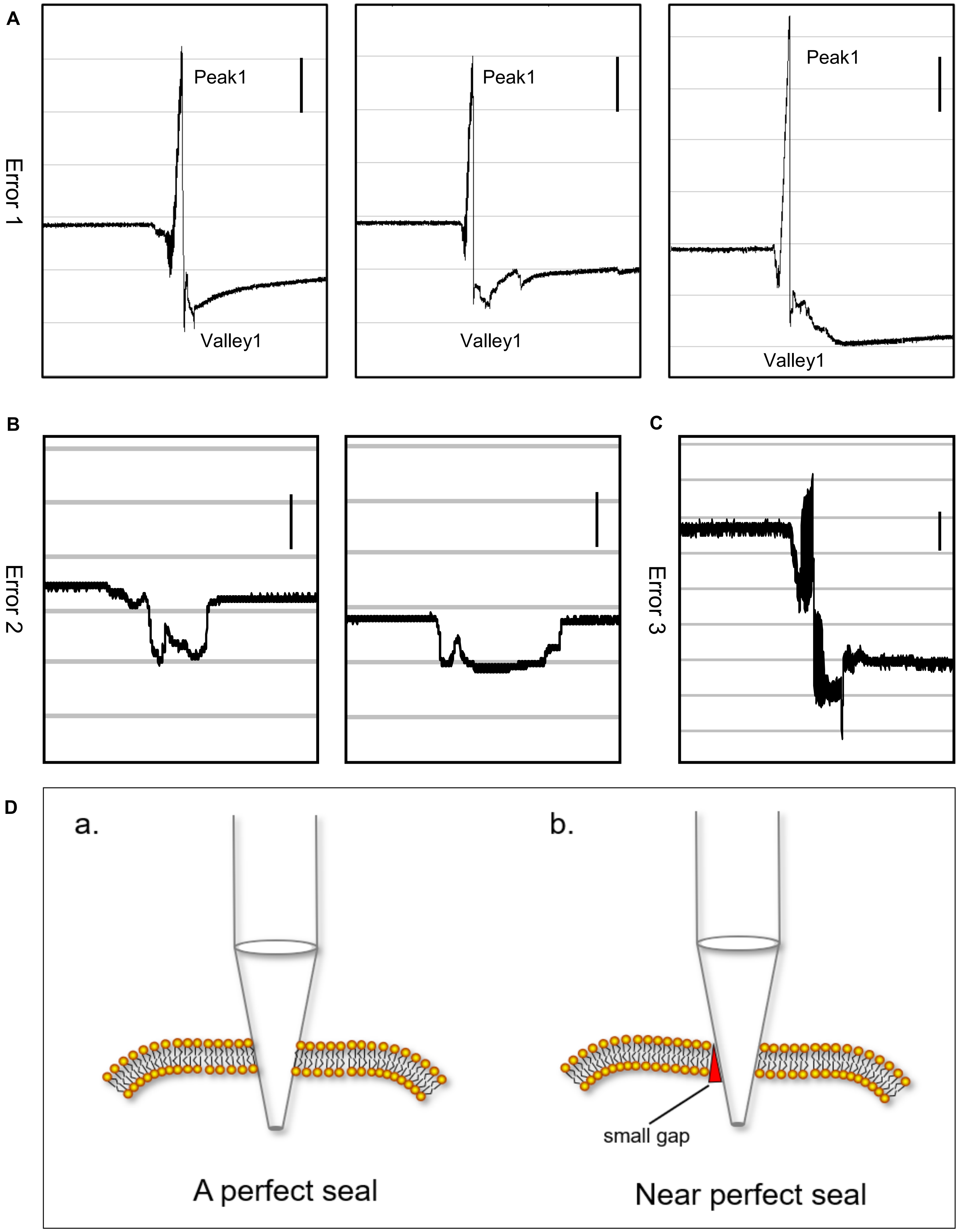

Figure S3. Unconventional potential shape.

A-C, Common error (1-3) transmembrane potential diagrams in *Arabidopsis*, bar = 5 mV.

D. Schematic diagram of electrode sealing to cell membrane, R-type (a) and r-type (b).

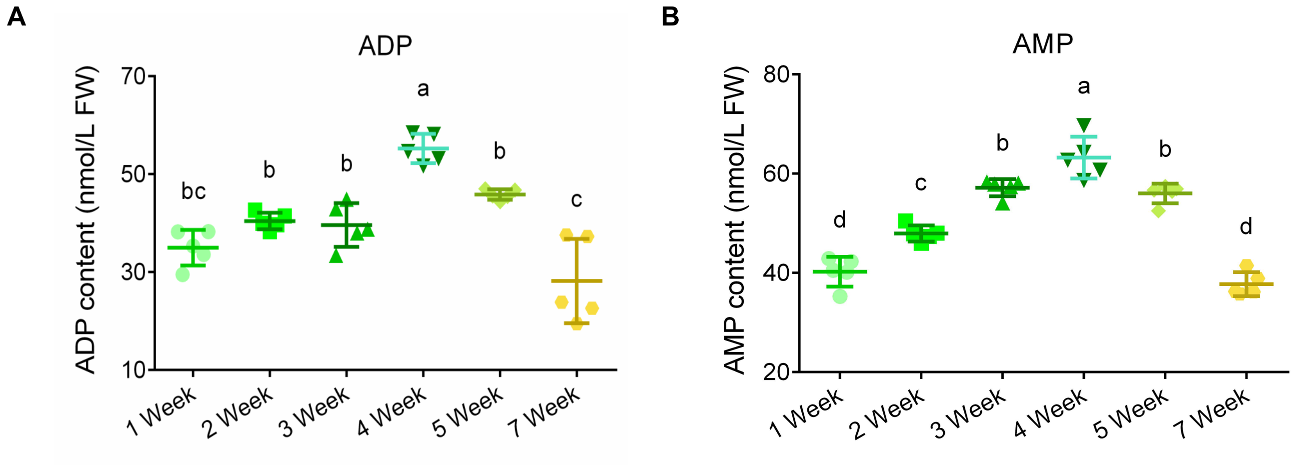

Figure S4. Contents of ADP and AMP at different growth stages.

Endogenous ADP (A) and AMP (B) contents of *Arabidopsis* rosette leaves at different growth stages. Error bars represent ± S.D., determined by one-way *ANOVA*.

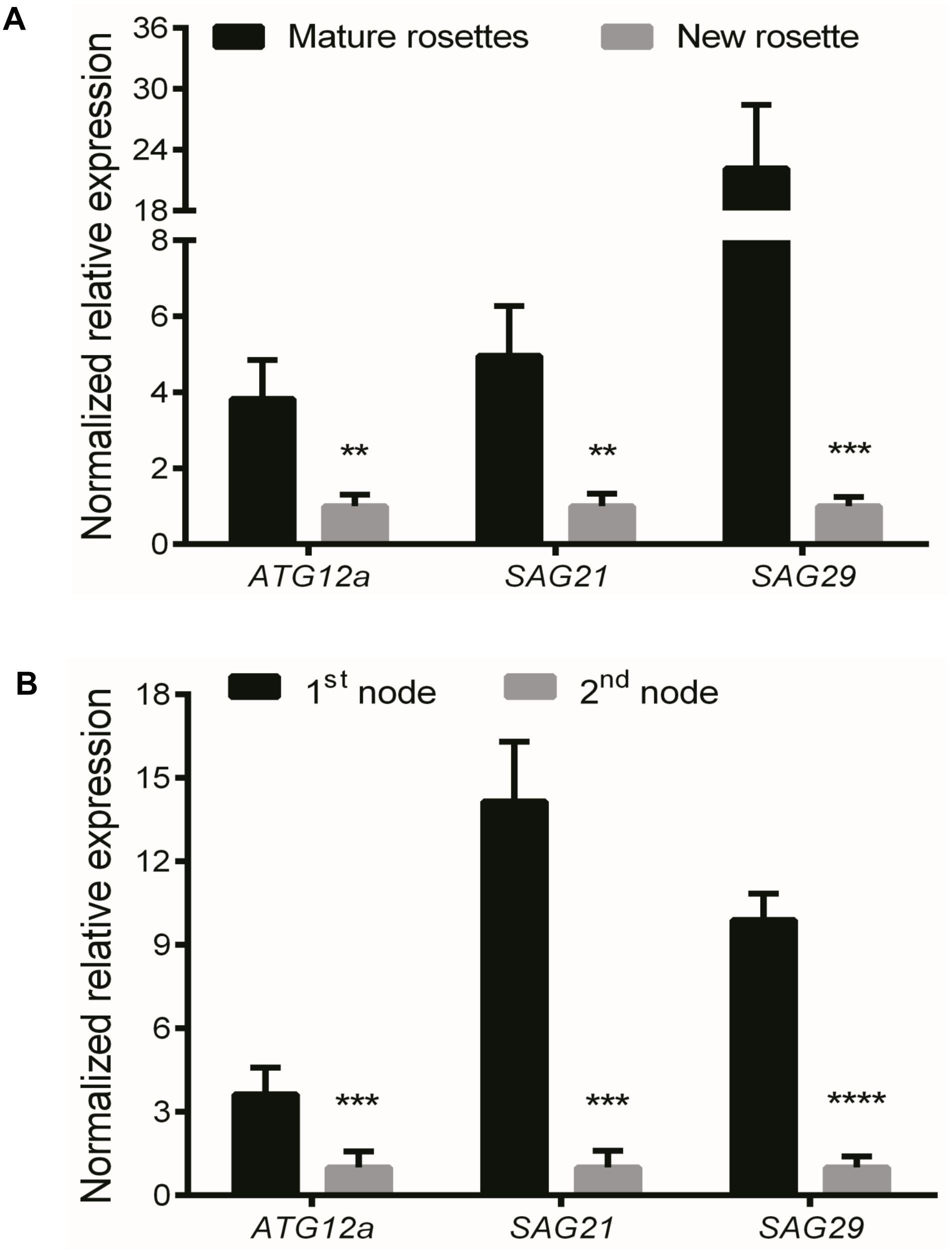

Figure S5. Expression of senescence genes in different organs.

ATG12a, SAG21 and SAG29 expression level in mature rosettes and new rosettes (A), 1st and 2nd cauline leaves (B). Data are presented as means ± SEM (n = 3). ** $p < 0.01$, *** $p < 0.001$, **** $p < 0.0001$; 2-way *ANOVA*.

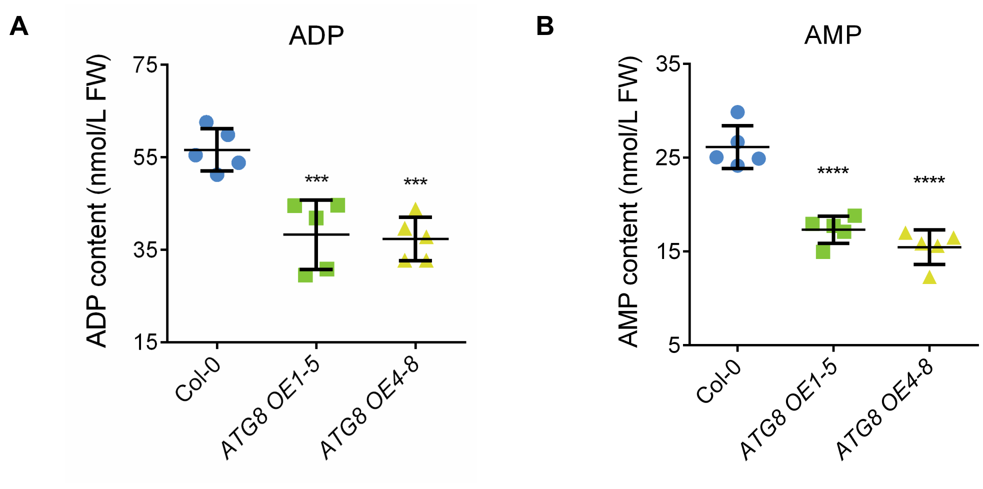

Figure S6. Contents of ADP and AMP in Col-0 and ATG8-OEs.

Endogenous ADP(A) and AMP (B) contents of *Arabidopsis* rosette leaves in Col-0 and ATG8-OEs. Error bars represent ± S.D., determined by one-way *ANOVA*.

Table S1. Primers used in this study.

**Primer name**	**Seuqence**	**Purpose**
qATG8-F	GCTTGGAGCTGAAAAAGCCA	RT-PCR
qATG8-R	GGATCAGACGTAGAAGCTGAGG	RT-PCR
qATG12-F	TCTCGCCAAACCCTGATGAA	RT-PCR
qATG12-R	TCCATTGCTTGGTTATGTACAGC	RT-PCR
qSAG21-F	GCGGCGACAAGAAGCTACAA	RT-PCR
qSAG21-R	TCATCACAGCCGAAGCAACA	RT-PCR
qSAG29-F	GAGTACATGCCCTTCACGCT	RT-PCR
qSAG29-R	CGACACACCTAACGGACTCA	RT-PCR
qUBQ5-F	GAAGATCCAAGACAAGGAAGGA	RT-PCR
qUBQ5-R	CTTCTTCCTCTTCTTAGCACCA	RT-PCR
